# Neuroactive steroids in the programming of neurodevelopment: implications of maternal obesity

**DOI:** 10.3389/fendo.2025.1703103

**Published:** 2025-11-17

**Authors:** Carmen J. Zamora-Sánchez, Juan Carlos González-Orozco, Jonatan Mendoza-Ortega, Mariana L. Villegas-Soto, Ignacio Camacho-Arroyo, Guadalupe Estrada-Gutierrez

**Affiliations:** 1 Department of Immunobiochemistry, National Institute of Perinatology, Mexico, Mexico; 2 Department of Genetics, The University of Texas MD Anderson Cancer Center, Houston, TX, United States; 3 Departamento de Inmunología, Escuela Nacional de Ciencias Biológicas, Instituto Politécnico Nacional, Ciudad de México, Mexico; 4 Unidad de Investigación en Reproducción Humana, Instituto Nacional de Perinatología-Facultad de Química, Universidad Nacional Autónoma de México (UNAM), Mexico, Mexico

**Keywords:** neuroplacentology, neurosteroids, pregnancy, fetal programming, allopregnanolone, steroid metabolism, body mass index, maternal obesity

## Abstract

Neuroactive steroids synthesized within the maternal-placental-fetal unit play a crucial role in fetal neurodevelopment by regulating cell proliferation, migration, and myelination, neurogenesis, gliogenesis, and synaptogenesis, ultimately shaping brain maturation. Dysregulation of neuroactive steroid metabolism, receptor signaling, and downstream pathways has been linked to neurodevelopmental and mood disorders. Maternal overweight and obesity, increasingly prevalent worldwide, induce profound metabolic and hormonal adaptations that may interfere with neuroactive steroid synthesis and function. These disturbances are associated with a higher risk of autism spectrum disorder, attention deficit hyperactivity disorder, and cognitive impairments in offspring, frequently with sex-specific effects. Despite these observations, the impact of obesity on neuroactive steroid levels and their regulatory roles during pregnancy remains poorly understood. This review synthesizes preclinical and clinical evidence on the biosynthesis, mechanisms of action, and neurodevelopmental effects of neuroactive steroids during the critical window of fetal programming. Furthermore, it highlights a current knowledge on how maternal overweight and obesity alter neuroactive steroid metabolism within the maternal–placental–fetal unit and explores their potential contribution to adverse neurodevelopmental outcomes. Addressing these knowledge gaps may uncover novel biomarkers and therapeutic targets to improve neurodevelopmental trajectories in populations increasingly exposed to maternal metabolic comorbidities.

## 1 Introduction

The terms *neuroactive steroid* and *neurosteroid* are frequently used interchangeably, although they differ in origin. Neurosteroids are synthesized locally within the nervous system, whereas neuroactive steroids encompass a broader group of steroids produced in peripheral tissues —including the gonads and adrenal glands— that exert regulatory effects on the central nervous system (CNS). Thus, all neurosteroids are neuroactive steroids, but not all neuroactive steroids are neurosteroids. This review focuses on neuroactive steroids in general, since, as we describe further, during pregnancy, these molecules are produced by distinct organs for both the mother and fetus.

During pregnancy, the maternal–placental–fetal unit, comprising the ovaries, placenta, and fetal adrenal zone, becomes a critical source of neuroactive steroids (1). These molecules interact with diverse receptors — including nuclear receptors, G-protein coupled receptors, membrane receptors, and ligand-gated ion channels (2–7)—modulating fundamental processes such as neurogenesis, synaptogenesis, myelination, and neuroprotection (1, 5, 7–10).

Neurodevelopment itself is a highly orchestrated process in which proliferation, migration, and differentiation of neural cells give rise to the cellular diversity and structural complexity of the CNS (11). The most vulnerable period spans “the first 1,000 days of life”, from conception through the first two years of postnatal life, during which, neuroactive steroids, such as progestogens, estrogens, androgens, and glucocorticoids are synthesized at particularly high levels by the maternal-placental-fetal unit. These steroid networks are highly sensitive to physiological, metabolic, and environmental influences. Among these, maternal overweight and obesity stand out as conditions that disrupt endocrine homeostasis, potentially altering the production and activity of neuroactive steroids during pregnancy.

Overweight and obesity are pathological conditions characterized not only by excessive fat mass but also by complex metabolic and endocrine disturbances. As adipose tissue functions as an active endocrine organ, obesity alters circulating adipokines, insulin sensitivity, and steroid hormone levels. Disruptions in these systems are known to affect fetal neurodevelopment and have been consistently associated with increased risks of autism spectrum disorder (ASD), attention deficit hyperactivity disorder (ADHD), and cognitive impairments in offspring. Importantly, alterations in neuroactive steroid levels—particularly allopregnanolone (3α-THP)—have been observed in neurodevelopmental conditions such as ASD (12, 13).

Despite these associations, direct evidence linking maternal obesity to disrupted neuroactive steroid synthesis or signaling remains scarce. This review addresses this gap by integrating mechanistic, preclinical, and clinical data to evaluate how maternal obesity modulates neuroactive steroids within the maternal–placental–fetal unit and how these alterations may shape neurodevelopmental trajectories.

## 2 Sources and metabolism of neuroactive steroids during prenatal and early postnatal life

Neuroactive steroids are key regulators of function and CNS homeostasis. Their influence begins in prenatal life and extends throughout the lifespan, with particularly critical roles during early neurodevelopment when neuronal circuits are formed and refined (14, 15). In mammals, neuroactive steroids reach the fetal brain via three main routes: maternal circulation, placental synthesis, and local fetal production—adrenal, hepatic, and gonadal tissues, as well as by the CNS (16). Here, we primarily focus on their synthesis within the developing brain, which originates from preexisting cholesterol, locally synthesized cholesterol, or sulfate-conjugated derivatives. As early as two weeks of gestation in humans, these steroid precursors are taken up by the placenta from the maternal circulation and transported to the fetus via various lipoprotein receptors, ATP-binding cassette transporters, and solute carrier transporters (17, 18). However, the placenta is not only involved in the uptake and transport of steroid precursors but also develops its own steroidogenic machinery. This activity becomes evident in the syncytiotrophoblast, which produces substantial placental neuroactive steroids, such as progesterone (P4), emerging between the 6th and 8th weeks of gestation in humans (19–21).

The synthesis of cholesterol in the prenatal brain (as well as in other common steroidogenic tissues) begins with two molecules of acetyl-CoA that are condensed to form acetoacetyl-CoA, which is then combined with another acetyl-CoA to produce 3-hydroxy-3-methylglutaryl-CoA (HMG-CoA). This molecule is reduced to mevalonate by the enzyme HMG-CoA reductase, the rate-limiting step of cholesterol synthesis. Subsequently, mevalonate is modified to produce isopentenyl pyrophosphate (IPP), which is finally converted to the direct precursors of cholesterol, squalene, and lanosterol (22). Although the mammalian embryo produces most of its own cholesterol, the maternal cholesterol supply is essential during the first weeks of life (23). However, once the blood-brain barrier is well established, the emerging CNS (particularly the brain) relies solely on *de novo* synthesis, as it is not permeable to circulating lipoprotein-bound cholesterol (24). This occurs at the embryonic stage E11-E12 in rodents and at 14 weeks of gestation in humans (16).

Once available, cholesterol is transported to mitochondria by proteins such as the 18 kDa translocator protein (TSPO) and the steroidogenic acute regulatory protein (StAR) (25). In the mitochondria, the P450 side-chain cleavage enzyme (P450scc, also known as CYP11A1) converts cholesterol to pregnenolone, which is already considered a neurosteroid (26). Studies have shown that the mammalian CNS can start to synthesize pregnenolone from the very early stages of prenatal development. In rodents, the expression of P450scc in neural tissue is detected as early as the E9.5 embryonic stage, while in humans, its expression is noticeable around the 10th week of gestation (5, 27). Besides local synthesis in the prenatal brain, other steroidogenic organs, such as the placenta, adrenal glands, and gonads, are also important sources of pregnenolone for the prenatal and early postnatal CNS (28), as the blood-brain barrier is easily permeable to this steroid (29). Upon availability of pregnenolone, it can be further metabolized into various steroids that play important roles in neurodevelopment. Pregnenolone is directly converted to P4 by the enzyme 3β-hydroxysteroid dehydrogenase (3β-HSD) (30). Since P4 is indispensable for pregnancy maintenance in mammals, the fetal CNS is continuously exposed to high levels of this steroid hormone from the maternal placental circulation. In humans, chorionic gonadotropin is produced early in pregnancy by trophoblasts to sustain corpus luteum-mediated P4 production during the first nine weeks of gestation. Afterward, the trophoblasts of the placenta take over this function (31, 32). In contrast, in rodents, the ovarian corpus luteum maintains P4 production throughout the entire pregnancy (31, 32). In addition to its luteotropic role, human chorionic gonadotropin also modulates hypothalamic signaling, contributing to the regulation of both the hypothalamic-pituitary-adrenal (HPA) and hypothalamic-pituitary-gonadal (HPG) axes in the mother and fetus (31, 32).

Regarding the local synthesis of P4, the human CNS produces this hormone from prenatal stages through postnatal life. Expression and activity of 3β-HSD, the key enzyme involved in P4 synthesis, have been detected in the fetal brain as early as the second trimester of pregnancy (33, 34). Importantly, prenatal and early postnatal local synthesis of P4 in the CNS is also noticeable in rodents and sheep. In rodents, in particular, the cerebellum exhibits the largest production of P4 within the CNS during the first days of neonatal life (35–38).

P4 is further metabolized into several neuroactive steroid derivatives. It is commonly converted by the 5α-reductase (5α-R) enzyme into 5α-dihydroprogesterone (DHP), which is later reduced to allopregnanolone (3α-THP) by the 3α-hydroxysteroid oxidoreductases (3α-HSD). 3α-THP is a neurosteroid with key roles in neurodevelopment (39). High levels of 3α-THP are observed during prenatal development and immediately after birth in species such as guinea pigs, sheep, and rodents. During late pregnancy, the CNS exhibits an increase in P450scc and 5α-reductase levels, suggesting that its capacity for synthesizing P4 and 3α-THP peaks around the time of birth (36, 38, 40, 41).

The presence and activity of androgens and estrogens have also been observed during early neurodevelopment. Pregnenolone is also a direct source of androgens. It is converted into the androgen dehydroepiandrosterone (DHEA) by the enzyme 17α-hydroxylase (CYP17A1); moreover, CYP17A1 also catalyzes the conversion of P4 into androstenedione (30, 42). Notably, the expression of CYP17A1 has been detected in the rat brain during prenatal development, and it decreases after birth (36). However, the adrenal cortex becomes an essential source of DHEA, a testosterone (T) precursor, during the first years of postnatal life (43, 44). Moreover, peripheral DHEA can cross the blood-brain barrier and become a source of other active androgens and estrogens in the CNS (45).

Besides its role in progestogen metabolism, 3β-HSD also converts DHEA into androstenedione. Then, the activity of the different 17β-hydroxysteroid dehydrogenases (17β-HSD) catalyzes the synthesis of T from androstenedione. Notably, 17β-HSD can also catalyze the conversion of DHEA into androstenediol, which is also used for T synthesis by the enzyme 3β-HSD (41). Then, T is further metabolized by 5α-R to dihydrotestosterone (DHT), which is a more potent androgen (46). Expression of 17β-HSD can be detected in the human placenta as early as the 4th week of pregnancy, which supplies fetal circulation with T and other active estrogens (20, 32). More importantly, the human fetal brain expresses and shows the activity of 17β-HSD enzymes as early as 13 weeks of gestation (47), while in mice, its expression is noticeable along with 5α-R expression starting at E12. Furthermore, 17β-HSD and 5α-R expression levels increase in the mouse fetal brain of males and females as development progresses (48).

As mentioned before, the placenta also supplies the fetal brain with estrogens, as it has been found in humans and rodents (20, 32, 49, 50). However, the mammalian CNS is also capable of local estrogen synthesis. It expresses the enzyme aromatase (CYP19A1) during prenatal and postnatal life in both sexes. The enzyme aromatase unidirectionally converts T into estradiol (E2). Nevertheless, it also catalyzes the conversion of androstenedione into estrone, which is further metabolized to E2 by 17β-HSD (51–54).

**Figure 1:**
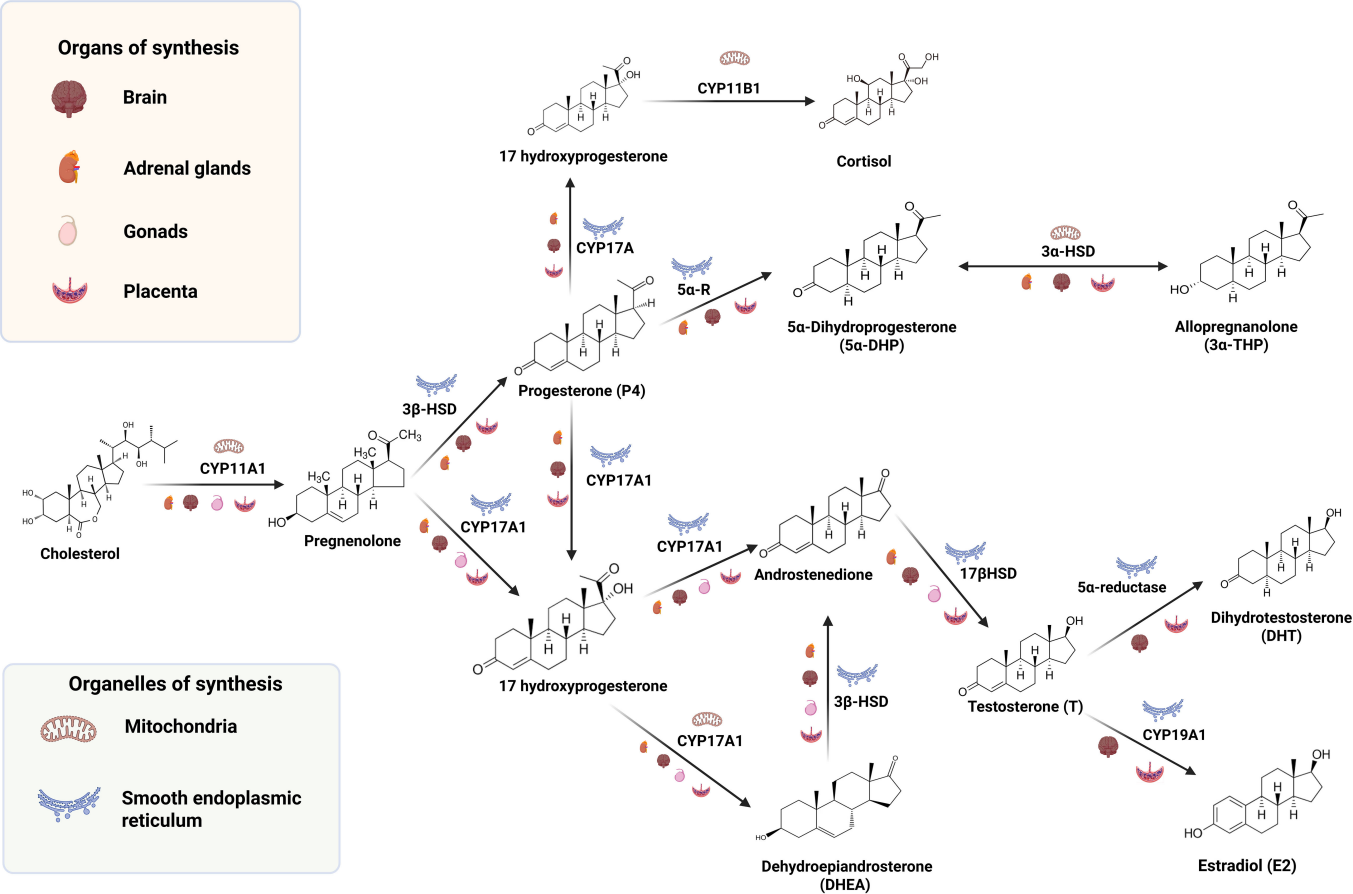
Schematic overview of steroidogenesis within the maternal–placental–fetal unit and developing brain, highlighting enzymes, subcellular compartments, and tissue sources relevant to prenatal and early postnatal neurodevelopment.

Glucocorticoids also play a key role during early neurodevelopment. Corticosterone and cortisol are the most relevant neuroactive steroids belonging to this group, and the adrenal cortex is the main source of these neuroactive steroids in the organism, which easily cross the blood-brain barrier (55). These neurosteroids are synthesized from P4 and its metabolite 17-OH progesterone. Specifically, the enzyme 21-hydroxylase (CYP21) synthesizes 11-deoxycorticosterone and 11-deoxycortisol from P4 and 17-OH progesterone, respectively. Then, P450C11 (CYP11) produces corticosterone and cortisol, respectively, from such substrates (41). In humans, maternal cortisol plays a major role and is easily transferred to the fetus via the placental circulation during the first trimester of pregnancy (56). After that period, the fetal adrenals can produce corticosterone and cortisol from maternal P4 starting at 8–10 weeks post-conception (57). Furthermore, after birth, maternal milk provides corticosterone and cortisol to the neonate (58). Besides, the postnatal CNS is also capable of glucocorticoid synthesis (55). Importantly, some authors have proposed that steroidogenic enzymes expressed in the fetal unit or in the placenta play a critical role in shaping the offspring’s development and behavior by turning steroid hormones into more polar compounds, unable to bind to nuclear receptors and modulating, instead, other type of receptors (59), as will be discussed in the next section. **Figure 1** summarizes a general scheme of steroidogenesis in the CNS as well as different tissues of the maternal-placental-fetal unit.

## 3 Neuroactive steroids: mechanisms of action and effects on neurodevelopment

Steroids with neuroactive effects exert their functions through very complex and diverse action pathways. These include both classical and non-classical mechanisms of steroid hormones, which are mediated by intracellular and membrane receptors. Intracellular or classical receptors act as transcription factors regulated by ligand that modulate gene expression, usually over a period of hours to days. These mechanisms influence cellular processes such as cell proliferation, differentiation, and metabolism, among others (5, 8, 42). In contrast, non-classical or non-genomic mechanisms depend on the activation of membrane-bound receptors and activate signal transduction pathways in a time span of seconds to minutes. This leads to changes in cellular functions, membrane cell polarization, calcium influx, and protein phosphorylation (60, 61). Lastly, these non-classical mechanisms can modify gene expression by regulating the activation of transcription factors downstream of signaling cascades. In addition, neuroactive steroids also modulate neurotransmitter receptors. Some of the most studied neurotransmitter receptors include GABA(A) receptors, NMDA, and AMPA receptors, which lead to changes in neural excitability. More recently, it has been proposed that, due to their lipophilic structure, neuroactive steroids regulate the fluidity of cell membranes and receptor trafficking (62). Moreover, crosstalk between steroid mechanisms could occur, as described further in this section.

Intracellular receptors involved in the neuroactive steroids signal transduction are progesterone (PR, isoforms: PR-A, and PR-B), glucocorticoid (GR, with several splice variant isoforms, such as GRα-γ), androgen (AR, with different splice variant), estrogen (ER, subtypes coded in different genes: ERα, and ERβ), and mineralocorticoid receptor (MR, with several splice variant isoforms, including MRα and MRβ). These are part of the nuclear receptor superfamily of transcription factors. Once activated by their ligands, they undergo a conformational change, promoting their release from chaperone proteins and facilitating their dimerization and translocation to the nucleus, where they bind to specific regions of DNA called hormone response elements, to recruit co-regulators, chromatin remodelers, and the transcription machinery [for review (63, 64)]. Besides its ligand activation, the activity and degradation of these proteins via the proteasome are regulated by post-translational modifications, such as phosphorylation, sumoylation, and ubiquitination (6, 65, 66). They also present non-classical mechanisms when located at the lipid rafts of the cell membrane or interacting with caveolin 1, or due to palmitoylation, which enables their polyproline-rich motif at the N-terminal domain of the receptor to interact with other proteins, such as the SRC family kinases (SFK). This interaction leads to the activation of several signaling cascades mediated by MAPK or Jak/STAT pathways (6). Such mechanisms have been described for PR (6), GR (64, 67), AR (4), ER (68), and potentially for MR (69). Although the latter has been better characterized by its interaction with receptor tyrosine kinases such as EGFR, and PDGFR, along with protein-coupled receptors like the G Protein-Coupled Estrogen Receptor 1 (GPER1, also known as GPR30) (70, 71). Steroid receptors and their mechanisms play a crucial role in differentiation, proliferation, and myelination of the nervous system, as well as brain plasticity throughout the lifespan. However, this is more relevant during the fetal and early neonatal periods as discussed forward.

In different types of tissues, including the human fetal tissues, PR-B has been widely associated with higher transcriptional activity, whereas PR-A has more transcription-inhibitory effects, particularly on the PR-B, ERβ, and GR (72). PR-A and PR-B have been identified in oligodendrocyte precursor cells derived from E14.5 mouse embryonic spinal cords, where P4 promotes proliferation in a PR-B–dependent manner. This has been demonstrated in cultured oligodendrocyte progenitor cells treated with the PR agonist R5020, in which silencing of PR-B abolishes the proliferative effect. Also, in these cells, P4 increases the expression of oligodendrocyte progenitor cell markers such as SOX9, NG2, while also inducing the expression of mature oligodendrocyte markers such as MBP and CNP1 (73), indicating that P4 induces oligodendrocyte maturation and myelination. *In vitro* assays show that along the differentiation process of mouse embryonic stem cells to dopaminergic neurons, they present higher expression of PR, while the expression of Erα decreases over the differentiation process (74). Moreover, the activation of ERα and dopamine D1 receptors induces the overexpression of PR-A in several brain areas of rat neonates (75, 76). This effect correlates with an increase in social play later in life, during the juvenile stage. Additionally, activation of PR and ERα promotes the differentiation of mouse embryonic cells to motoneurons, as demonstrated by *in vitro* experiments using ER subtype antagonists (8).

In humans, ERβ and GR are highly expressed in human undifferentiated embryonic stem cells and embryoid bodies, compared to Erα (77). GR was also highly expressed in embryoid bodies and the three developmental layers (77). Besides, they have also been identified in several brain areas of chicks, rats, and mice from embryonic to early postnatal life66. In murine models, the expression of ERα and ERβ has been demonstrated to be related to sexual differentiation of the brain. The expression of ERα has been enriched in brain areas related to sexual behavior, such as the hypothalamus, while the expression of ERβ is more broadly expressed throughout the brain [for review about ER effects on neurodevelopment and its effects on behavior, see (78)]. Besides, the estrogen, estetrol, a highly produced steroid during pregnancy, has antioxidant, antiapoptotic, and pro-myelinating effects mediated through ERα and ERβ (79).

Corticosteroids influence neurodevelopment in fetal and early postnatal life through the activation of neurogenesis in a GR-dependent manner and an MR-dependent manner. The GR expression has been reported in all cell types of human cerebral organoids from induced pluripotent stem cells. In such models, treatment with dexamethasone, a GR agonist, enriches the expression of transcription factors associated with neural differentiation like *PAX6, FABP7*, and *NEUROD1* (80). However, it must be mentioned that cortisol at high doses could negatively impact the neurogenesis of dopaminergic neurons at the ventral tegmental nucleus in males but not females (81). Although the key role of glucocorticoids and the GR has been highlighted, it is also important to consider that excessive glucocorticoid levels, such as those seen in excessive maternal stress or obesity, can have detrimental effects on neurodevelopment (82), as we will explore in the next sections.

Regarding MR, this receptor plays a role in the antenatal development of the hippocampus. Its expression is detected in specific fetal brain regions, such as the hippocampus and hypothalamus, at embryonic day E16.5 in rats, shortly before the end of gestation at E22.5 (83). Other animal models, such as guinea pigs, display a different pattern, with MR expression decreasing in the same brain regions near delivery, highlighting a markedly species-dependent regulation of MR expression (84, 85).

Along with the ERs, the AR plays a key role in the sexual dimorphism of the brain, particularly in the brain cortex and the arcuate nucleus of the hypothalamus. *In vitro* studies in human neural stem cells, treatment with the potent androgen, DHT induced cell proliferation and survival when cells were exposed to nutrient-deprived conditions (86). Additionally, in these cells, DHT upregulated the expression of genes related to ASD (NRCAM, FAM107A, IGFBP5). In humans, RNA-Seq data indicate that the expression of AR in the brain cortex decreases over time from 8 weeks postconceptional to 15–17 weeks postconception (wpc) in both 46XY and 46XX fetuses. Notably, the expression of the 5α-R1 is augmented over the same development period in both males’ and females’ brain cortex. In this study, Buonocore et al. focused on this critical period because of the beginning of testicular production of T at 8 wpc in XY individuals. In such individuals, higher differences were seen in the brain cortex gene expression, attributed to chromosomal differences and Carnegie developmental stages, rather than due to steroid production by the fetal testis (87). Nonetheless, this must be further studied.

Apart from the AR, rapid effect exerted at the cell membrane, several proteins have been proposed as membrane androgen receptors, such as the transient receptor potential melastatin 8 (TRPM8), a calcium channel, or the G protein-coupled receptors GPRC6A, and the Oxoeicosanoid receptor 1 (OXER1), to which T present high affinity and exhibit effects in cancer cells (88). In animal models, their expression has been observed in hippocampal neurons and seems to be crucial for learning and memory through the activation of ERK kinases (89).

Additionally, membrane receptors mediate rapid mechanisms of neuroactive steroids, although they are less understood than those of intracellular receptors. Some of the most studied of these receptors are the five membrane P4 receptors: mPRα, mPRβ, mPRγ, mPRδ, and mPRϵ. They are located at cellular membranes and transduce signals through G-protein activation (60). Also, P4 membrane receptor components 1 and 2 (PGRMC1/2) are membrane receptors located in proximity to mPR and regulate its activity. Such receptors are activated by P4 and its metabolites with different affinities depending on the structure of each mPR type (60). These receptors are differentially expressed in fetal neural tissue, with mPRα and mPRβ being the most highly expressed (90). They mediate the activation of cAMP/PKA, PI3K/AKT, and MAP kinase/ERK1/2, by which mPRs induce neurite outgrowth (90). In zebrafish, mPRγ is involved in neural proliferation, particularly in the olfactory system and the brain shape (91). They also mediate the anti-apoptotic effects of 3α-THP and its synthetic analogs in cell models lacking intracellular receptors, by interacting with the GABA(A) receptor, the main neurotransmitter receptor regulated by neuroactive steroids, whose mechanism of action will be discussed further below (92).

Estrogens also regulate fetal neural development through the membrane receptor (GPER) (61), which is highly expressed in the rodent brain, particularly in the adult brain. However, there are very few studies about their relevance in fetal neurodevelopment, especially in humans. Similarly to mPRs, GPER activates G proteins and mediates neurite, axon, and dendrite growth in E18 hippocampal mouse neurons (93, 94). In mice, GPER participates in preventing neural cell death, astrocyte viability, and expression of inflammatory cytokines when cells are treated with high doses of glutamate (88, 95).

The GABA(A) receptor is a ligand-gated ion channel regulated by neuroactive steroids (96, 97). Its chemical structure is critical in determining the type of regulation that neuroactive steroids will exert on GABA(A) receptor. For example, 3α-steroid metabolites positively modulate it, while 3β-steroid metabolites negatively regulate it (98). The most important positive regulators of GABA(A) receptor are 3α-THP, allotetrahydrodeoxycorticosterone, androstanediol, and 17α-OH allopregnanolone (99). In human neural stem cells and rat hippocampal neuroprogenitor cells, the co-expression of GABA(A) receptor and the Na^+^-K^+^-2Cl^-^ cotransporter SLC12A2, promotes the efflux of Cl^-^, which induces depolarization of the cell membrane, and the subsequent activation of voltage-dependent L-type calcium channels, the activation of PKA kinases, and the expression of cell cycle regulators, lastly inducing proliferation (98, 100, 101). Through these mechanisms, GABA(A) receptors and neuroactive steroids promote the increase in cell number in proliferative brain zones such as the embryonic cortex and the ventricular zone (101–103).

P4 plays a crucial role in sustaining pregnancy. Research involving pregnant rats shows that maternal P4 can cross the fetal bloodstream and affect the developing CNS. Also, as previously mentioned, progestogen neuroactive steroids begin to be synthesized in the placenta and locally in the fetal nervous system from the very early stages of life (5). Therefore, it has been suggested that P4 and its close derivative, 3α-THP, are crucial for proper neurodevelopment in mammals, serving specific functions in this process (1, 5). For several years, it has been noted that P4 has neuroprotective effects in the CNS, and its therapeutic use has even been clinically investigated for conditions such as traumatic brain injury (TBI), spinal cord injury (SCI), and epilepsy (104–107).

In the context of neurodevelopment, it is noteworthy that the newly formed CNS is highly vulnerable to injury from intrauterine complications, neonatal hypoxia-ischemia, and excitotoxicity, leading to neuronal death and impaired neurodevelopment. This can result in permanent neurological deficits and neonatal death (31, 106, 108). In this way, P4 and 3α-THP have been shown to mitigate CNS damage in rats and sheep shortly before and after birth by regulating cell proliferation and reducing inflammation and apoptosis during traumatic injury events and birth asphyxia (96, 97, 106, 109). Furthermore, in newborn rats, P4 treatment lessened inflammation, with fewer activated microglia in the cortex and hippocampus following hypoxia-induced brain injury. Treated pups also showed better motor and cognitive recovery by three weeks post-injury (106). Similarly, in newborn mice, P4 reduced inflammation and induced neuronal survival, suggesting that its effects involve regulating pro-inflammatory cytokines (108, 110). Interestingly, disruption of 3α-THP synthesis in sheep fetuses increases apoptosis and astrocyte proliferation in the brain (3, 96). Given that GABA is an inhibitory neurotransmitter, 3α-THP is believed to maintain a suppressive state in fetal brain neuronal activity during late gestation to prevent excitotoxicity, as demonstrated in sheep. This effect dampens fetal brain activity and helps to regulate fetal sleep-like behaviors, which are crucial for proper neurodevelopment (40, 111). Excitatory effects of GABA(A) receptor have also been characterized in neural progenitors of rats and humans, in which 3α-THP induces hyperpolarization of the cell membrane and induces cell proliferation (100, 112, 113). Besides, 3α-THP promotes the migration and maturation of Schwann cells through its modulatory effect on GABA(A) receptor and the activation of SRC and FAK kinases (114, 115).

Apart from its neuroprotective and neuromodulatory effects, 3α-THP is also strongly associated with birth outcomes in humans and with the neurodevelopment of the newborn in an indirect manner, since pregnancy is characterized by an increase in the levels of P4 and 3α-THP in the maternal plasma due to the placental synthesis (1, 9). A decrease in these neuroactive steroids after delivery has been associated with the development of the maternal psychiatric condition known as postpartum depression (116, 117), which also impacts the behavioral and cognitive development of the newborn (118). Importantly, it has also been shown that placental 3α-THP insufficiency in mice leads to cerebellar myelination impairment after birth, which correlated with autistic-like behavior in male offspring (119). P4 has been involved in neurodevelopmental myelination, particularly in the early postnatal cerebellum of rodents (120, 121). It is also reported that P4 could be involved in oligodendrocyte differentiation in the mouse embryonic spinal cord (73). Likewise, P4 increases mature oligodendrocyte survival after white matter injury induced by chronic hypoxia in neonatal rats (122).

Androgens have been largely associated with brain development, particularly in the first and second decades of life in humans. The evidence shows that androgens, especially DHEA, play a crucial role in brain cortex maturation in an age- and sex-specific manner during middle childhood and adolescence (123). Interestingly, this group of neuroactive steroids also has effects during fetal and early postnatal stages of neurodevelopment. One of the key roles in fetal neurodevelopment is its involvement in brain sexual differentiation, as high levels of this androgen contribute to brain masculinization (124). Additionally, T and its immediate metabolite, DHT, have been shown to influence the maturation of hippocampal neural circuits in a sex-specific manner during the first few postnatal days in rats. Notably, T also contributes to the masculinization of this brain structure since its neonatal administration to females masculinizes spatial memory performance, a function largely associated with the hippocampal circuitry (125–127). The effects of T and DHT on cortical and hippocampal maturation are presumed to be due to the regulation of neurogenesis, neurite growth, and synaptic density (128, 129). In support of this, it has been demonstrated that DHT upregulates the expression of genes associated with proliferation and cell survival in human embryonic neural stem cells (86).

In recent years, growing evidence has positively linked androgens to the early onset of human neurodevelopmental conditions. Particularly, ADHD and autism have been related to prenatal exposure to high levels of T, since these conditions are more frequent in males (86, 130–132). Since T and DHT are involved in synaptic density and neural circuitry maturation, it is hypothesized that their high levels in the maternal-fetal circulation dysregulate gene expression associated with the neuronal events, leading to an excitation-inhibition imbalance in the developing brain (133).

Regarding estrogens’ actions during early neurodevelopment, E2 has been shown to play a critical role in brain development and function, influencing neurogenesis, synaptogenesis, and neuroprotection (134). The enzyme aromatase is widely expressed in the forebrain of primates (including humans), and although estrogens are more associated with female functions, the expression of this enzyme in the CNS does not depend on plasma E2 levels (135, 136). Moreover, E2 levels and aromatase expression in the fetal brain have been associated with adequate cortical development in rats and mice (134, 137). Particularly, evidence from ERβ knockout mice shows that E2 influences the migration and survival of cortical neurons at late prenatal stages (138). Also, *in vitro* studies have shown that E2 promotes the proliferation of neural stem cells isolated from rat embryonic telencephalon and oligodendroglial cells (139). Additionally, the knockdown of ERβ in mouse embryonic neural stem cells reduces the number of progenitors of mesencephalic dopaminergic neurons that originate from them (140). Likewise, E2, through the GPER, stimulates neurite outgrowth in hippocampal neurons from the E17 mouse fetal brain (141), indicating that it also plays a role in neurogenesis and the maturation of neural circuits in different brain structures.

To make masculinization effective at the molecular and cellular levels, aromatization of T to E2 is required (142). Then, E2, through the ER, changes gene expression patterns to influence the number of neurons and shape of dendritic spines in the most sexually dimorphic brain regions, including the preoptic area and the hippocampus (143, 144). Importantly, Vacher et al. recently reviewed the significance of sex steroid levels, especially androgens, and their association with neurodevelopmental conditions such as autism in different cohorts. In such studies, higher levels of androgens in mothers with polycystic ovaries were associated with a higher risk of autism in males. Also, they remark on the relevance of neuroactive steroids synthesized by the placenta at the very early developmental stages as major regulators of neurodevelopmental processes (1).

Glucocorticoids exert key functions in the CNS and play a fundamental role in pregnancy at late stages. Maintaining glucocorticoid levels is so important that they are administered to women at risk of preterm delivery, since it favors pulmonary maturation (145, 146). Besides, premature birth is often associated with a disruption of proper neurodevelopment, leading to conditions such as cognitive impairment, cerebral palsy, and speech deficits (147). Notably, maternal cortisol and corticosterone disruption have been related to these conditions as they regulate neurogenesis, neuronal migration, plasticity, and neurotransmitter activity (147, 148). It has been reported that physiological levels of cortisol stimulate the proliferation of human hippocampal neural progenitors, while high levels associated with stress conditions can inhibit the differentiation of these cells (149). In the same way, elevated levels of corticosterone due to a stress response affect the proliferation and differentiation of neural stem cells during embryonic and adult neurogenesis (150, 151). Additionally, it has been suggested that high levels of maternal glucocorticoids associated with stress conditions during pregnancy affect proper neurodevelopment by altering the maturation of neural circuits, impacting the individual’s behavior after birth and throughout life (152–154).

**Figure 2:**
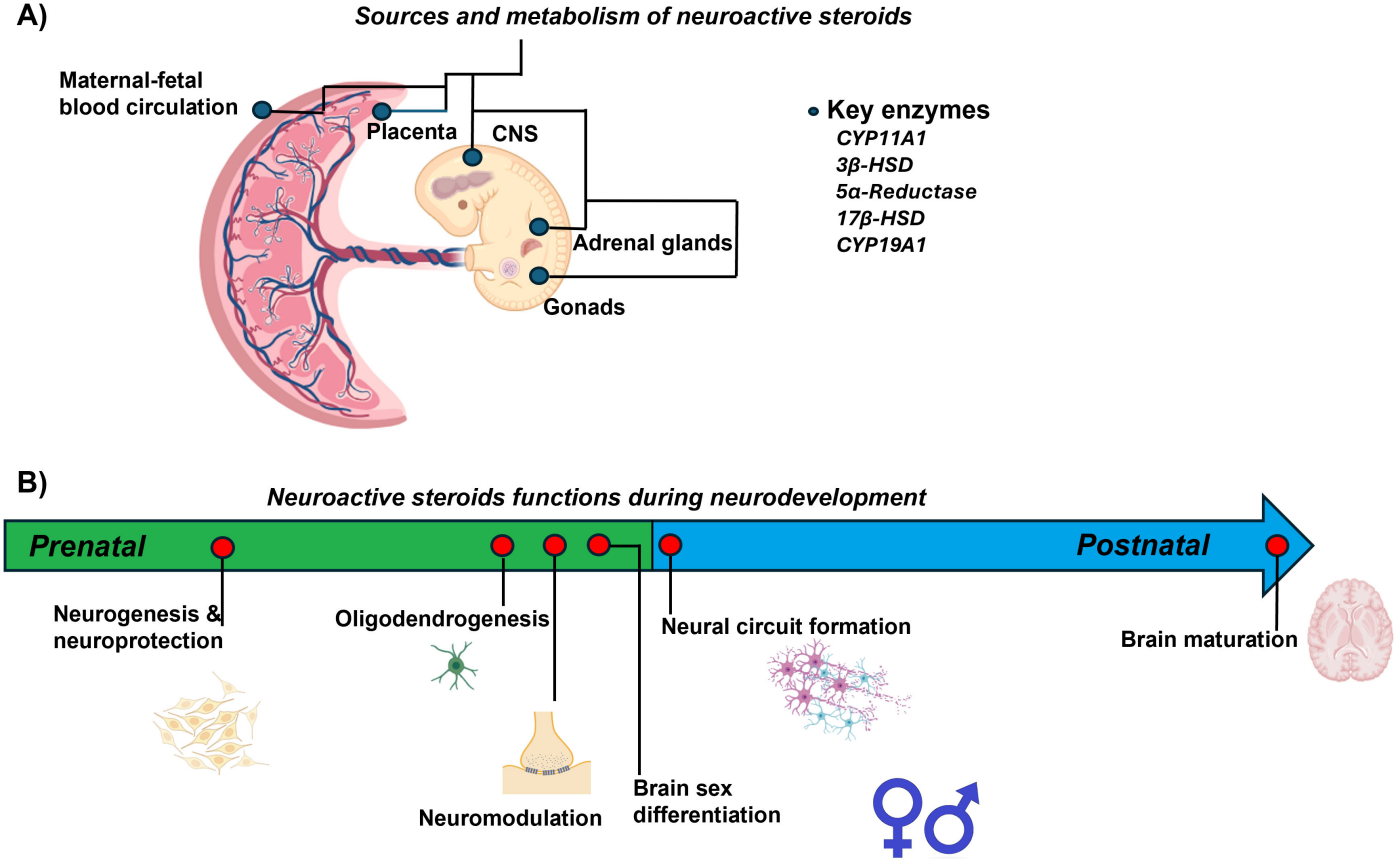
Summary of neuroactive steroid actions during prenatal and early postnatal neurodevelopment. **(A)** Sources and metabolism of neuroactive steroids in the maternal-placental-fetal unit. **(B)** Neuroactive steroids function during neurodevelopment. The arrow indicates events regulated by neuroactive steroids during fetal and early postnatal life.

All functions mediated by neuroactive steroids, highlight the relevance of these molecules in understanding how their dysregulation, regardless of the underlying cause, could compromise the proper development of the CNS. **Figure 2** presents a summary of the most important biological events regulated by neuroactive steroids involved in neurodevelopment during prenatal and early postnatal life. In the following section, we will discuss in greater detail how obesity and overweight can alter neurosteroid action and, consequently, affect early CNS development.

## 4 Neurodevelopmental outcomes in offspring of mothers with overweight and obesity

Maternal overweight and obesity impact the neuroendocrine, metabolic, and inflammatory regulation systems in women. These conditions, particularly during pregnancy, predispose to adverse reproductive health outcomes, including infertility, gestational hypertension, preeclampsia, gestational diabetes, postpartum hemorrhage, emergency cesarean deliveries, and both intrauterine growth restriction and macrosomia. Overweight and obesity in pregnant women can influence the developing fetal CNS through various mechanisms, encompassing hormonal, metabolic, inflammatory, and epigenetic pathways, leading to early adverse CNS development outcomes and mood disorders in their offspring (155–158). Outcomes differ depending on whether the exposure is pregestational obesity or excessive gestational weight gain.

### 4.1 Outcomes associated with pregestational overweight and obesity

Preclinical models show that maternal obesity prior to conception alters fetal brain structure, connectivity, and behavior. Studies in mice have shown that pregestational obesity leads to a reduction in hippocampal cortical thickness and an increase in the soma and branches of astrocytes in the offspring (159). Other studies hypothesize that obesity in the offspring is predisposed by pregestational maternal obesity due to changes in the food-intake circuits innervation. In this line, offspring of pregestational obese female mice present less innervation of neuropeptide Y neurons to the paraventricular nucleus and less neurite growth in the arcuate nucleus of the hypothalamus at gestational day 17.5 compared to offspring of pregestational normal weight mothers. Such nuclei are critical hubs for adipokine signaling of hunger and satiety (160). Also, the offspring of these mothers showed a significantly increased density of GFAP-positive astrocytes in the arcuate and supraoptic nuclei of the fetal hypothalamus. This increase may indicate disrupted hypothalamic programming induced by the intrauterine inflammatory environment (161). In addition, mouse offspring of pregestational obese females present early deficits in communication and olfactory discrimination, impaired social play behavior, and reduced expression of synaptopodin, a protein involved in synaptic plasticity in the hippocampus and the prefrontal cortex (162).

In rats, maternal pregestational obesity has been associated with an increased incidence of congenital CNS malformations, including neural tube defects and microcephaly. These conditions reflect disruptions in early neurodevelopment (163). Besides, elevated glucose levels and insulin resistance, which are usual comorbidities along with obesity, have also been reported to alter brain development and neuronal maturation in rats (164). Also, in these animals, obesogenic diets have been shown to cause deficits in cognitive functions along with damage in the hippocampus and the prefrontal cortex (165). It is important to consider the time of exposure to this obesogenic diet, for example, several months before pregnancy vs only during pregnancy, also have different effects, which are explored in other reviews (165).

Pregestational overweight and obesity in humans have been more consistently linked to structural and functional alterations, including increased rates of behavioral symptoms, emotional dysregulation, and psychiatric disorders. Maternal pregestational body weight is also associated with the development of the autonomic nervous system, evaluated *in utero* by measuring the heart rate variability at trimesters 1 and 3. In this study, the heart rate variability was inversely correlated with the maternal body weight (166). High maternal BMI at the beginning of pregnancy has also been associated with poor inter-hemispheric communication, poor functional connectivity between the prefrontal cortex and the anterior/inferior insula of the left frontal gyrus in the fetal brain (167), and reduced cortical thickness in key neonatal brain regions, such as the left frontal lobe (168).

Pregestational maternal obesity has also been reported to impair skills such as memory, attention, inhibitory control, and problem-solving (164). Also, some studies have linked maternal pregestational overweight and obesity with lower cognitive, linguistic, and motor skill scores in children at 2 years of age (169, 170). A meta-analysis of 41 studies from 36 perinatal cohorts—including populations from the United States, the Netherlands, the United Kingdom, Denmark, Finland, Sweden, Australia, and Norway—reported that children of women with pregestational overweight had a 17% higher likelihood of neurodevelopmental disorders, while those of women with obesity showed a 51% increased risk compared to children of mothers with normal weight (171). More specifically, the meta-analysis identified elevated risks for attention-deficit/hyperactivity disorder (62%), autism spectrum disorder (36%), cognitive delays (58%), and emotional or behavioral problems (42%). Other cohort studies have linked maternal obesity before conception to lower performance in cognitive domains such as practical reasoning at 36 months of age (153) and verbal recognition at 9 years (172).

In summary, there are global and homogenous data of preclinical and clinical studies indicating that maternal pregestational overweight and obesity alter signaling, connectivity, and CNS structure during fetal development, and increase the risk of different neurodevelopmental conditions such as ADHD, autism spectrum disorders (ASD), eating disorders, anxiety, and depression in their offspring later in life (156, 164, 165). In **Table 1**, we present a summary of clinical studies regarding the effects of pregestational overweight and obesity on anatomical changes and poor neurodevelopmental outcomes. These findings consistently indicate that maternal pregestational overweight or obesity are associated with adverse neurodevelopmental outcomes in offspring. Overall, elevated maternal adiposity appears to affect brain structure, cognitive performance, and behavioral regulation during early life and childhood, potentially through inflammatory, metabolic, and microbiome-related pathways. However, heterogeneity in study design, sample size, and neurodevelopmental assessments, along with differences in the age of participants in these studies, limits the comparability of existing data. Future research should aim to delineate critical windows of vulnerability, elucidate the underlying biological mechanisms, and determine whether targeted nutritional or metabolic interventions during pregnancy can attenuate these adverse outcomes.

**Table 1:** Summary of clinical studies on neurodevelopmental outcomes in offspring of mothers with pregestational overweight or obesity.

Country (year)	Experimental design	Sample of dyads	Outcomes in the offspring of women with overweight and obesity	Ref.
U.S. (2021)	Prospective cohort study. Healthy full-term 2-week-old neonates underwent brain magnetic resonance imaging during natural sleep. All women had their body composition measured at 12 weeks of pregnancy.	44	↓ Cortical thickness in the left pars opercularis gyrus, left pars triangularis gyrus, and left rostral middle frontal gyrus. Negative correlation between maternal body fat mass percentage and mean cortical thickness in these frontal lobe regions.	(168)
Spain (2022)	Prospective case-control study. Neuroanatomical differences were assessed in children aged 6 years. Dyads were divided into six groups based on their calculated maternal p-BMI at the recruiting visit and the diagnosis of GD at 34 weeks of gestation.	143	Neuroanatomical differences were observed only in children born to mothers with both GD and pregestational overweight. Brain differences appeared to reflect global alterations rather than focal changes in specific cortical or subcortical regions.	(216)
Spain (2023)	Prospective case-control study. Mothers were divided into four groups according to their p-BMI and their GD status. Children were evaluated with Bayley III scales of neurodevelopment at 6 months and 18 months.	331	At 6 months: ↑ scores in cognition, composite language, and expressive language in children of women with obesity. At 18 months: They lost points in composite language scores, and the previous differences in language and cognition were replaced by a suggestive trend of lower gross motor scores in children of women with obesity.	(217)
U.S. (2019)	Prospective cohort. Child (age 7) ADHD symptoms were assessed using the Child Behavior Checklist subscale and for neurocognitive function using the Go/No-go task and correlated with maternal p-BMI and GWG.	172	↑Scores for the Go/No-go task, indicating less efficient processing (obese vs normal only weight). Maternal GWG was not significantly associated with performance. Maternal p-BMI was significantly associated with child ADHD symptoms.	(218)
France (2022)	Prospective cohort of children of mothers recruited in early gestation. Hyperactive-inattention symptoms were assessed using the Strengths and Difficulties Questionnaire at 3, 5, and 8 years.	1307, 1184, and 875 at 3, 5, and 8 years, respectively	Positive association with increased odds of the offspring having high Hyperactive-inattention symptoms between 3 and 8 years old.	(219)
U.S. (2016)	Prospective cohort. Grouped by self-reported maternal pregestational obesity and diabetes. ASD and developmental disorders in children were based on physician diagnoses documented in electronic medical records.	2734	Maternal pregestational obesity and pregestational diabetes in combination were associated with ↑ risk of ASD and Intellectual disabilities.	(220)
Italy (2021)	Prospective cohort. The Griffiths Mental Developmental Scales and the Extended revised version were used to measure children´s cognitive development in age ranges of 6–24 and 36–60 months. Microbiota composition was assessed by 16S rRNA in first-pass meconium samples and in stool samples collected at 3, 6, 12, and 36 months in children.	90	Overweight correlates with offspring gut microbiota composition and cognitive development in the practical reasoning domain at 36 months of age.	(221)
U.S. (2017)	Prospective cohort. p-BMI was self-reported. At ages 9–11 years, children were evaluated with the Peabody Picture Vocabulary Test (receptive vocabulary) and the Raven Coloured Progressive Matrices Tests (perceptual reasoning)	2,084	Pregestational overweight and obesity were associated with lower scores for the Peabody Picture Vocabulary Test.	(172)
Finland (2021)	Prospective cohort study. Neurodevelopment was assessed with the Bayley Scales of Infant and Toddler Development-Third Edition, and the Hammersmith Infant Neurological Examination. Maternal adiposity was determined by air displacement plethysmography, p-BMI, and GD with an oral glucose test. Dietary assessment included diet quality and fish consumption questionnaires, and three-day food diaries.	243	GD was associated with weaker expressive language skills. Higher maternal adiposity was associated with weaker cognitive, language, and motor skills in children. Higher fish consumption was related to better expressive language skills.	(169)
U.S. (2016)	Prospective cohort study. Mothers completed a questionnaire about health status and lifestyle, both parents’ height and weight, p-BMI, total GWG, and weight at delivery. Parents completed the Ages and Stages Questionnaire when their children were 4, 8, 12, 18, 24, 30, and 36 months of age, corrected for gestation.	5034	↑ Odds of failing the fine motor domain. Children whose parents had BMI >35 were likely to fail the problem-solving domain additionally.	(170)

p-BMI, pregestational body mass index. GD, gestational diabetes. ADHD, attention deficit hyperactivity disorder. GWG, gestational weight gain.

### 4.2 Outcomes associated with excessive gestational weight gain

Gestational obesity is typically reflected by weight gain above Institute of Medicine guidelines. Few information has been described regarding neurodevelopmental outcomes and excessive weight gain during pregnancy, which is a complex condition, influenced not only by an increased food intake and a poor quality of diet, but also includes pregestational overweight, frequent cravings, less exercise, and psychosocial factors such as depression and body image dissatisfaction (173–175). Excessive weight has been mostly associated with outcomes related to body composition rather than neurodevelopmental disorders. Regarding body composition, the most consistent studies have demonstrated the association of excessive weight gain and the outcomes of large-for-gestational age and macrosomia (157, 176–178). About neurodevelopmental outcomes, most studies consistently show a positive association between excessive weight gain and ASD, as summarized in **Table 2**. Clinical evidence from multiple prospective and case–control studies suggests that excessive gestational weight gain (GWG) and maternal overweight or obesity during pregnancy are consistently associated with an increased risk of adverse neurodevelopmental outcomes in offspring, as summarized in **Table 2**. Reported outcomes include a higher incidence of global neurodevelopmental disorders (NDD), autism spectrum disorder (ASD), attention deficit hyperactivity disorder (ADHD), and intellectual developmental disorder (IDD), as well as subtle impairments in cognition, language, and executive functioning. Excessive GWG—particularly when superimposed on pregestational overweight or obesity—emerges as a consistent and independent risk factor for suboptimal neurodevelopmental outcomes. Although causality cannot yet be firmly established, the reproducibility of associations across diverse populations and study designs highlights the potential importance of gestational weight management as a modifiable target for the prevention of neurodevelopmental disorders. Future longitudinal and mechanistic studies integrating metabolic, inflammatory, and neuroimaging biomarkers are needed to elucidate causal pathways and identify critical windows for effective intervention.

**Table 2:** Summary of clinical studies on neurodevelopment outcomes in the offspring of mothers with gestational overweight and obesity.

Country (year)	Experimental design	Sample of dyads	Observed outcomes in the offspring of women with overweight and obesity	Ref.
Sweden (2023)	Prospective cohort study. Children were followed from 2 to an average of 7.4 years old. NDD diagnoses were ascertained using information gathered from all potential care pathways in Stockholm County. GWG z-scores and RGWG (kg/week) were obtained from the Obstetrics record system.	57,822 children born to 53,516 mothers	12% increased risk of any neurodevelopmental disorders. Higher RGWG was associated with a 28% increased risk of NDD diagnosis.	(222)
U.S. (2016)	Prospective cohort study. Interviews over the second and/or third trimesters, delivery weight via medical chart review, and infant neurobehavioural exams (NICU Network Neurobehavioural Scale) at 2 days and 32 days postpartum were done.	261	Poorer self-regulation (less active and greater irritability).	(223)
Canada (2023)	Prospective cohort study. Maternal p-BMI was self-reported, GWG was estimated. Child neuropsychological assessments included intelligence, language, memory, motor skills, executive function of inhibitory control, and behavior.	379	Only GWG’s above recommendations were associated with lower scores on the Phonological Processing and Narrative Memory subtests.	(224)
U.S. (2015)	Prospective cohort study. Dyads followed from early pregnancy to 10 years postpartum. IQ was assessed using the Stanford Binet Intelligence Scale-4th edition. Executive function was assessed by the number of persevering errors on the Wisconsin Card Sorting Test and time to complete Part B on the Trail Making Test. Self-reported total GWG was converted to gestational-age-standardized GWG z-score.	763	↓ Offspring IQ [3.2 points] and a slower time to complete the executive function scale Part B. High GWG (>+1SD) is positively associated with a slower executive function task.	(184)
U.S. (2016)	Prospective cohort study. Self-reported total GWG was converted to gestational-age standarised z-scores. Child ADHD symptoms were assessed with the Conners´ Continuous Performance Test. Child behavior was assessed by parent and teacher ratings on the Child Behavior Checklist and Teacher Report Form.	511	Maternal GWG was correlated with offspring impulsivity errors depending on p-BMI.	(225)
Spain (2022)	Prospective cohort study. Singleton births of >22 weeks of gestation of women with GD. Groups considered p-BMI and excessive GWG.	1036	Among GD pregnancies, maternal pregestational obesity with excessive GWG was associated with the highest risk of ADHD.	(226)
Sweden (2021)	Prospective cohort study. Mothers were classified by GWG. Offspring´s intellectual developmental disorders were extracted from the Swedish National Patient Register.	467,485	Excessive GWG had higher risks of ID. Extremely excessive GWG (>25 kg) increased offspring´s IDD risk only among mothers with an early p-BMI>25 kg/m^2^.	(227)
Sweden (2015)	Prospective cohort study. ASD diagnosis was ascertained covering all potential pathways to ASD care and diagnosis in Stockholm County.	333,057 children of 176,860 mothers	Excessive GWG had higher risk of ASD.	(228)
Lebanon (2022)	Case-control study using a random proportional sample of ASD to explore risk factors by applying a questionnaire.	66 ASD and 66 control children	A higher GWG was significantly associated with higher odds of ASD.	(229)
Iran (2022)	Case-control study. Data was collected using medical records and face-to-face interviews with mothers.	208 ASD and 218 control children	Maternal GWG was significantly higher in the ASD group.	(230)

NDD, neurodevelopmental disorder. GWG, gestational weight gain. RGWG, rates of gestational weight gain. P-BMI, pregestational body mass index. ADHD, attention deficit hyperactivity disorder. GD, gestational diabetes. IDD, intellectual developmental disorder. ASD, autism spectrum disorder.

In animals, high-calorie, or high-fat diets administered only during pregnancy are often used as models of gestational obesity. In mice, a gestational obesity model fed from the preimplantation to lactation with a high-fat diet, induced a higher density of immature neurons in males and a lower density of mature neurons in the dentate gyrus with an increased density of astrocytes and microglia in the hippocampus of both male and female adult offspring (179). In rats, this type of diet induces in the offspring, atrophy in hippocampal pyramidal cells, primarily affecting basal dendrites, while apical dendrites remain largely unaffected. It is suggested that elevated leptin levels during early development contribute to the observed dendritic morphological alterations, as leptin plays a neurotrophic role in brain development (180). In this model, gestational obesity also impairs memory, as evaluated by conditioned pain aversion assays in the offspring (180).

### 4.3 Neurotransmitter signaling pathways affected by maternal obesity

Some of the mechanisms altered by maternal obesity are related to dopaminergic and serotonergic (5-HT) signaling systems. In mice, sex-specific responses in the regulation of dopamine-related genes in fetuses have been found. Male fetuses showed increased expression of the dopamine receptor D2 (DRD2) in the endocannabinoid system within the prefrontal cortex, without changes in dopamine metabolites. In female fetuses, DRD2 expression decreased while homovanilic acid, the final product of dopamine metabolism, increased, suggesting enhanced dopamine metabolism (181). In another study, mice exposed to a high food diet *in utero* demonstrated dysregulation of the dopamine reuptake transporter, which is associated with impairments in eating behaviors and reward response to food, and its association with schizophrenia, ASD, and ADHD has also been demonstrated (155).

Regarding the role of 5-HT system in offspring of obese women, it has been shown that a high-fat diet reduces 5-HT synthesis in offspring, associated with reduced serotonergic axonal density and embryonic neuronal survival in human brain regions critical for behavioral regulation, and contributes to an increased risk of neurodevelopmental pathologies such as ADHD, ASD, anxiety, and depression. In mice, it has been described that maternal high-fat diet induces an increase in the offspring hippocampal levels of the enzyme TPH2 involved in 5-HT synthesis. Besides, in humans, methylation of the 5-HT receptor 2A gene (HTR2A) promoter, has been associated con with psychiatric conditions such as schizophrenia and bipolar disorder effects in four analyzed cytosines in the promoter region, located – 166 (L1), - 1439 (L2), -1421 (L3) and –1224 (L4), particularly some specific genetic polymorphisms (rs6311 and rs6306). It has been reported that higher pregestational BMI is associated with lower HTR2A promoter methylation in placentas of male fetuses of the (182).

An additional indicator of synaptic overexcitation is glutamate levels, which are also affected by maternal obesity, with increased concentrations observed in mouse fetuses in the endocannabinoid system within the prefrontal cortex (181). Furthermore, maternal high-fat diets have been shown to alter fetal brain glutamate metabolism. In this context, levels of glutathione disulfide (GSSG)—the oxidized form of glutathione that also interacts with NMDA and AMPA glutamatergic receptors—are decreased in fetal brains of mothers that received a high-fat diet. This reduction may have behavioral implications, given that glutamate is the main excitatory neurotransmitter in the CNS (183). In the same study, the expression of *Tbr1*—a gene typically expressed by excitatory glutamatergic neurons in the amygdala and involved in regulating the expression of neuronal activation markers—was decreased in response to maternal high-fat diets (184). This is further supported by a mice model study, which reported that in the context of maternal high-fat diets, Mecp2 was detected in the amygdala of male offspring and was associated with upregulation of Tbr1. Tbr1 is normally repressed by Mecp2 during brain development, a regulatory mechanism that is critical for proper neuronal differentiation and neurite formation. Mutations or dysregulation of Mecp2 have been linked to several neurodevelopmental disorders. In line with this repressive role, previous studies indicate that reduced Mecp2 activity leads to increased Tbr1 transcription. Consistent with these findings, the authors observed elevated Tbr1 mRNA levels and a higher number of Tbr1-expressing cells within the basomedial (BM) nucleus of the basolateral amygdala in offspring of mothers that received a high-fat diet (185). Additionally, the expression of glutamate receptors such as GluR7 and the glutamate ionotropic receptor NMDA type subunit 2A (Grin2a), as well as scaffolding proteins such as Shank2, critical for synaptic plasticity, are altered in such model of obesity (183). The increased baseline glutamatergic activity in the absence of behavioral stimuli suggests an imbalance in neuronal excitation, which should predispose offspring to behavioral alterations, particularly anxiety, ASD, and ADHD (183, 185).

Although pregestational and gestational obesity are distinct, they are often overlapping conditions. For example, women with overweight or obesity also tend to present excessive weight gain during gestation (173–175). As mentioned in this section, both factors could independently and additively increase the likelihood of adverse outcomes. However, pregestational obesity often has a stronger and more persistent effect on offspring’s health. Moreover, research about the effects of excessive weight gain during pregnancy is needed.

## 5 Insights into neuroactive steroid status in the maternal-placental-fetal unit in overweight and obesity

As mentioned before, beginning pregnancy with a condition of overweight or obesity, and its associated comorbidities such as hypertension, insulin resistance, type 2 diabetes, or polycystic ovary syndrome, are associated with an increased, chronic pro-inflammatory state and an altered metabolic environment, rather than a more acute state of metabolic adaptation to gestational obesity. There is an interplay between energy homeostasis and neuroactive steroids´ functions. In this line, overweight and obesity are conditions characterized by excessive energy storage, as mentioned before, and include alterations in the local and peripheral effects of neuroactive steroids. Importantly, neuroactive steroids are modulators of the hypothalamic-pituitary-adrenal and hypothalamic-pituitary-gonadal axes, as well as mood and sexual behavior modulators. They are also regulators of energy storage in adipose depots, homeostasis of reproductive function, among others, and altered levels and signaling of neuroactive steroids in female overweight and obesity have been broadly described (186–188). They also influence alterations in steroid hormone levels in the maternal-placental-fetal unit, as described in this section. Importantly, an emerging trend in research suggests a potential distinction in the neurodevelopmental outcomes associated with maternal obesity, depending on the timing of exposure: pregestational obesity or excessive weight gain during pregnancy, as mentioned before.

Maternal obesity is increasingly recognized as a state of chronic low-grade inflammation, or metainflammation, driven by altered metabolic and immune regulation (189, 190). During pregnancy, this inflammatory milieu is further exacerbated, exposing the fetus to an intrauterine environment enriched in cytokines and metabolic stressors that can disrupt key neurodevelopmental processes. Women with obesity frequently exhibit elevated levels of IL-6, IL-8, TNF-α, IFN-γ, and CRP, along with altered adipokine profiles, which can impair placental functions such as trophoblast invasion, nutrient transport, and cytokine signaling (190). These alterations compromise the maternal–fetal interface, allowing proinflammatory mediators to influence fetal brain development.

Neuroinflammation and oxidative stress have been implicated in microglial activation, lipid peroxidation, and cytokine overexpression in offspring (189). Inflammatory mediators such as pJNK and TNF-α can disrupt brain-derived neurotrophic factor (BDNF) metabolism and tryptophan hydroxylase-2 (TPH2) expression—key regulators of hippocampal neurogenesis and serotonin synthesis. Furthermore, maternal inflammation enhances placental serotonin production, which has been linked to impaired axonal growth and abnormal neuronal connectivity (189). It has also been suggested a complex interplay between immune signaling and neuroactive steroids, since steroid hormones such as P4, E2, T, cortisol, DHEA, and 3α-THP are key immunomodulators whose effects as pro- or anti-inflammatory often depend on their concentration (191–194). Collectively, these findings indicate that the maternal proinflammatory state in obesity reshapes placental signaling and fetal brain development, predisposing offspring to long-term neurobehavioral vulnerabilities. Neuroactive steroids may exert a bidirectional modulatory role that remains to be fully elucidated.

Research about the role of steroids in fetal programming is increasing, particularly in the new field of neuroplacentology, which studies how placental factors influence both normal and altered perinatal outcomes, such as preterm birth, fetal growth restriction, and associated poor neurodevelopmental outcomes (1, 119, 195). This is crucial for species adaptation because it has been shown that maternal steroid hormones are rapidly metabolized by fetal-placental tissues to shape the fetal brain and modulate neurodevelopment and behavior as an adaptive consequence to the exposure of maternal steroid hormones, and independently of its classical effects mediated by their nuclear steroid hormone receptors (1, 59, 196). Despite this, very few studies have focused directly on the influence of maternal overweight and obesity on the maternal-placental-fetal unit neuroactive steroids metabolism, their signaling and its consequences on fetal neurodevelopment.

Pregestational maternal overweight and obesity are linked to significant changes in fetal steroid hormone synthesis. A prospective cohort study of Lassance et al., which included 144 pregnant women with obesity and 90 normal weight ones, showed decreased plasmatic levels of P4 and E2 at 38.5–40 weeks of pregnancy prior to their scheduled C-section. Importantly, in this study, the mitochondrial fraction of the placentas showed significantly lower levels of the TSPO protein, involved in the cholesterol transport to the mitochondrion, a rate-limiting step in the biosynthesis of neuroactive steroids, along with lower cholesterol levels in the pregestational obesity group compared with the normal weight one (197). A case-control study was conducted to evaluate the umbilical cord metabolome of neonates born to mothers with either normal weight (n=32) or overweight/obesity (n=33). In this study, the levels of 23 steroid metabolites were significantly lower in the umbilical cord of neonates in the pregestational overweight/obesity group compared with normal weight mothers. Among the most relevant in terms of their potential as neuroactive steroids were T, 7α-hydroxytestosterone, corticosterone, 11-deoxycortisol, and cortisol (198). Moreover, in a study of 80 women presenting gestational diabetes mellitus (BMI < 25 Kg/m^2^: n = 56; BMI > 25 Kg/m^2^: n = 24) and 79 pregnant women without diabetes (BMI < 25 Kg/m^2^: n = 67; BMI > 25 Kg/m^2^: n = 25) at 23–26 gestational weeks^218^, the diabetes group presented lower serum levels of pregnenolone, P4, E2, and estrone than the normal-weight group. Besides, pregnant women with obesity and diabetes mellitus present a higher ratio of E3/E2 and a decreased ratio of estrone/androstenedione (199).

Glucocorticoids, particularly cortisol, are one of the most extensively studied neuroactive steroids associated with poor neurodevelopmental and behavioral consequences in the offspring. Obesity deregulates the maternal and fetal hypothalamic-pituitary-adrenal axis, contributing to altered levels of cortisol (155). Consequently, maternal obesity influences fetal brain development by augmenting fetal cortisol levels, particularly in male offspring in baboons (200). Cortisol concentrations may vary depending on the anatomical site and the species. However, evidence indicates that women with high BMI early in pregnancy present lower concentrations of cortisol and corticosterone in umbilical cord blood. This imbalance is linked to altered activity of the enzymes 11β-HSD1, which synthesize cortisol, and 11β-HSD2, which inactivates it, potentially affecting glucocorticoid transfer and metabolism in the placenta (201). Also, a systematic review found that maternal obesity reduces the activity of the placental 11β-HSD2, which diminishes fetal cortisol metabolism into a less active metabolite, cortisone. This reduction in cortisol metabolism may increase fetal exposure to cortisol (202) and alter the programming of the hypothalamic-pituitary-adrenal axis, impacting neuroendocrine development (202, 203). In preterm birth, which is highly associated with poor neurodevelopmental outcomes, cortisol levels are augmented. In a nested case-control study within the framework of the Healthy Start Study cohort with paired healthy controls and preterm delivery cases, there was a significant association with higher cortisol levels at weeks 13 to 22, and preterm birth (204).

As mentioned before, 3α-THP is associated with neurodevelopment and child behavior (1, 59). However, its metabolism is understudied in the context of pregestational obesity and neurodevelopmental outcomes in the offspring. In a study with 9 women diagnosed with polycystic ovary syndrome and 24 age-matched controls (16 with normal weight and 8 with overweight), it was observed that women with overweight and polycystic ovary syndrome presented higher 3α-THP concentrations than controls with normal-weight (205), suggesting that obesity modifies 3α-THP metabolism in women with comorbidities associated with obesity.

It is hypothesized that 3α-THP plays a role in the pathophysiology of obesity *per se* since it regulates GABAergic signaling involved in feeding regulation at the hypothalamus (206). In male rodents, P4 and 3α-THP induce food intake. This aspect has not been evaluated in female rodents along the estrous cycle (207, 208). Articles published in the 90s indicated that food intake increases in women during the luteal phase, which correlates with the period of the highest P4 and 3α-THP leves (206). However, in women with obesity, there are contradictory reports regarding this hypothesis about the role of 3α-THP in the etiology of obesity. Some articles have reported that 3α-THP levels are low in women with obesity, while T is elevated when compared to normal healthy ones (209), which is also related to a higher susceptibility for these women to present mood disorders such as anxiety or depression throughout life, including postpartum (118).

Notably, one study indicates that women with very low levels of 3α-THP beginning pregnancy or with high 3α-THP levels during the third trimester and with a rapid drop near birth have been associated with higher depressive symptoms at the postpartum (210). However, this study only includes women with a normal weight, so it is important to explore this in women with obesity. Conversely, another study found a negative correlation between 3α-THP maternal blood levels and BMI in the first trimester. This effect was also observed for women who smoked in the first trimester and those who developed pregnancy-induced hypertension (211). In this study, there was no correlation between 3α-THP levels and depressive symptoms. A Swedish longitudinal study of 56 women reported that 3α-THP determined at gestational weeks 12 and 35 was slightly positively correlated with the weight increase during pregnancy. In a group with higher weight gain (≥ 11 Kg) there were significant higher 3α-THP levels compared to the < 11 Kg group (212).

These data indicate that 3α-THP imbalances are related to maternal mood disorders, especially during pregnancy and postpartum. Research in this topic has led to the development and FDA approval of brexanolone, a pharmaceutical form of 3α-THP as a treatment for postpartum depression. Besides, other studies indicate that such imbalances in 3α-THP levels could be related to the weight composition of the mother (212).

Regarding the offspring’s health, little has been explored about the role of 3α-THP in dyads of women with pregestational overweight or obesity. Since preclinical studies have indicated that 3α-THP is involved in fetal neurodevelopment, some authors have hypothesized that low levels of 3α-THP during fetal life are related to a higher risk of ADHD, ASD disorders, or anxiety during childhood and adolescence (213). Some conditions that are associated with a lower fetal exposure to 3α-THP and other neuroactive steroids are high exposure to maternal stress, increased cortisol levels, or preterm birth (204). Although this hypothesis has been broadly evaluated in models such as guinea pigs (214, 215) it has not been evaluated in clinical research nor in the context of maternal pregestational obesity.

## 6 Conclusions

Maternal overweight and obesity clearly increase the risk of adverse neurodevelopmental and neuropsychiatric outcomes, including ASD, ADHD, cognitive delays, and mood disorders in offspring, which can persist throughout life. Neuroactive steroids—central regulators of neurogenesis, myelination, synaptic formation, and immune modulation—represent a plausible mechanistic link between maternal metabolic status and offspring brain development.

Although animal models provide strong evidence that maternal obesity alters steroidogenesis and receptor signaling, human data remain scarce and inconsistent, particularly regarding allopregnanolone and other pregnane derivatives. Addressing these gaps requires integrative approaches combining maternal endocrine profiling, placental and cord blood metabolomics, and longitudinal neurodevelopmental follow-up.

The emerging field of neuroplacentology provides a conceptual framework to explore how maternal metabolic conditions reprogram fetal neurodevelopment via placental steroid metabolism. Advancing this field could inform novel biomarkers, preventive strategies, and even therapeutic interventions to mitigate adverse neurodevelopmental trajectories in children exposed to maternal obesity.
